# The Relationship between Breakfast and Sleep and Cardiovascular Risk Factors

**DOI:** 10.3390/nu15214596

**Published:** 2023-10-29

**Authors:** Yejin Kim, Hye-Ji An, Young-Gyun Seo

**Affiliations:** Department of Family Medicine, Hallym University Sacred Heart Hospital, Anyang 14068, Republic of Korea; 4613052@naver.com (Y.K.); hjian9011@gmail.com (H.-J.A.)

**Keywords:** breakfast, sleep, obesity, hypertension, diabetes, dyslipidemia, metabolic syndrome, cardiovascular disease

## Abstract

Despite extensive research on the individual effects of breakfast and sleep on health outcomes, there has been limited investigation into their combined effects. We aimed to evaluate the relationship between breakfast-eating behavior and sleep timing on cardiovascular disease (CVD) risk factors. A total of 16,121 participants (6744 men and 9377 women) aged 19 years or older were selected from the Korea National Health and Nutrition Examination Surveys (2016–2018, 2021). We classified participants into four groups: early sleep + regular breakfast eaters (group 1), late sleep + regular breakfast eaters (group 2), early sleep + infrequent breakfast eaters (group 3), and late sleep + infrequent breakfast eaters (group 4). In men, group 4 had a lower prevalence of obesity than group 1 (OR 0.78, 95%CI 0.62–0.97), and groups 2, 3, and 4 had a higher prevalence of metabolic syndrome (MetS) than group 1 (OR 1.43, 1.62, and 1.47, respectively). In women, group 4 had a lower prevalence of dyslipidemia than group 1 (OR 0.59, 95%CI 0.44–0.80), and group 2 had a higher prevalence of MetS than group 1 (OR 1.24, 95%CI 1.03–1.50). The combination of skipping breakfast and late sleep timing was associated with the higher prevalence of MetS particularly in men. Moreover, the relationship between breakfast and sleep timing on CVD risk factors differed by sex and age group.

## 1. Introduction

Sleep–wake patterns and the timing of fasting-food intake are two modifiable lifestyle factors that make up the 24-hour cycle [1]. Breakfast, regarded as the most important meal of the day, is distinct from the other meals in that it breaks the longest overnight fast [2,3]. The health consequences of skipping breakfast have been the subject of research for decades, with a series of studies finding that it is associated with obesity, diabetes mellitus (DM), hypertension (HTN), and metabolic syndrome (MetS) [4,5,6,7], all of which are risk factors for cardiovascular disease (CVD). Sleep is another essential factor that affects our health and well-being [8]. Although previous research has mainly focused on the impact of sleep duration, sleep timing is a distinct characteristic of sleep that influences obesity and metabolic health independently of sleep duration [9,10,11]. The timing of sleep is crucial, as it may lead to circadian misalignment due to discordance between the 24-hour light–night cycle and lifestyle patterns [12]. Moreover, multiple studies have shown that a later bedtime was associated with an increased risk of CVD risk factors [13,14,15,16,17].

Sleep timing and breakfast are co-dependent. Later bedtime and chronotype were associated with late-night food intake, low-quality dietary habits, and skipping breakfast [18,19,20]. Furthermore, research using mendelian randomization has provided evidence of a causal relationship between evening chronotype and skipping breakfast, as well as genetically determined breakfast skipping and increased body mass index (BMI) [21]. Given the increasing evidence that combinations of risk factors may impact health outcomes differently than individual factors in isolation, it is important to understand how breakfast and sleep may interact and contribute to the development of CVD risk factors [22,23].

Despite extensive research on the individual effects of breakfast and sleep on health outcomes, there has been limited investigation into their combined effects. In this study, we hypothesized that the combination of breakfast and sleep timing (early sleep + regular breakfast, late sleep + regular breakfast, early sleep + infrequent breakfast, and late sleep + infrequent breakfast) would have different relationships with CVD risk factors. In particular, we predicted that skipping breakfast and late sleep might be associated with a higher incidence of CVD risk factors. Therefore, the aim of this study was to explore the joint relationship between breakfast-eating behavior and sleep timing on CVD risk factors, including obesity, DM, HTN, dyslipidemia (DL), and MetS, in Korean adults and to investigate whether the relationship varied by age and sex.

## 2. Materials and Methods

### 2.1. Study Design and Participants

The Korea National Health and Nutrition Examination Survey (KNHANES) has been performed by the Korea Disease Control and Prevention Agency (KDCA) since 1998 to produce representative statistics on the health and nutritional status of Koreans. 25,341 participants aged 19 years or older were selected from the seventh to eighth surveys (2016–2018, 2021; bedtime was investigated only for 4 years). Among them, 7544 were excluded according to the following exclusion criteria: missing test results or survey records; inadequate water intake (≥90 g/kg of body weight); inadequate nutritional intake (>5000 or <500 kcal/day); inadequate fasting time before test sampling (<8 or >24 h); renal dysfunction (estimated glomerular filtration rate <30); and a history of diagnosed cancer. Bedtimes from 7:00 AM to 6:59 PM, that is, data from 7:00 to 18:59, when the number of subjects by hours was less than 50, were also excluded (1676). Consequently, data from 16,121 participants (6744 men and 9377 women) comprised the final dataset.

All procedures were approved by the Ethics Committee of the KDCA (approval numbers 2018-01-03-P-A and 2018-01-03-3C-A) and were conducted in accordance with the ethical standards laid down in the 1964 Declaration of Helsinki and its later amendments. All KNHANES participants signed informed consent forms. The KNHANES data were made publicly available.

### 2.2. Sleep Timing

We used the following variables: bedtime during the weekdays, bedtime during the weekends, average hours of sleep per day on weekdays, and average hours of sleep per day on weekends. These were calculated based on two questions: 1. “On typical weekdays (or working days), at what time do you go to bed and wake up?” 2. “On typical weekends (or non-working days), at what time do you go to bed and wake up?” Respondents recorded specific sleep onset and waking times for both questions.

We calculated average weekly bedtime, average weekly sleep duration, mid-sleep on free days (MSF), and MSF corrected for sleep debt on workdays (MSFsc) using the following formula [24,25]:

average weekly bedtime = (bedtime during the weekdays ∗ 5 + bedtime during the weekends ∗ 2)/7

average weekly sleep duration = (average hours of sleep per day on weekdays ∗ 5 + average hours of sleep per day on weekends ∗ 2)/7

MSF = bedtime during the weekends + sleep duration during the weekends/2

MSFsc = MSF—(sleep duration during the weekends—average weekly sleep duration)/2

The range of time designated from 1:00 to 24:59 was changed to 0:01 to 24:00 (24 h were subtracted for times exceeding 24:00). Therefore, the range of MSFsc data used for analysis was from 0:01 to 24:00. Since bedtime continues from night to dawn, and sleeping at dawn means sleeping later than sleeping at night, data from 19:00 to 24:00 were used as-is, but data from 0:01 to 6:59 were changed to 24:01 to 30:59 by adding 24 h. Therefore, the range of bedtime data used for analysis was from 19:00 to 30:59. For consistency of expression, 24 h were subtracted for bedtimes exceeding 24:00 to change to the original time format after analysis.

The median bedtime of participants was 23:17, and we classified bedtimes as early sleep when the bedtime <23:17 and as late sleep when the bedtime ≥23:17.

### 2.3. Breakfast Eating Behavior and Group

We classified participants into different types of breakfast eaters using the following questions from the survey: “How many times a week did you eat breakfast in the past year?” Four response options were provided: 1. 5–7 times a week 2. 3–4 times a week 3. 1–2 times a week 4. Rarely (0 times a week). Participants who answered ‘1–2 times a week’ or ‘rarely (0 times a week)’ were classified as ‘infrequent breakfast eaters’ and those who answered ‘5–7 times a week’ were classified as ‘regular breakfast eaters’. Therefore, we classified participants into 4 groups according to sleep timing and breakfast eating behavior, as follows: early sleep + regular breakfast eaters (group 1), late sleep + regular breakfast eaters (group 2), early sleep + infrequent breakfast eaters (group 3), and late sleep + infrequent breakfast eaters (group 4).

### 2.4. Other Variables

We also used the following variables: age, daily nutritional intake (total energy, carbohydrate, protein, and fat intake), average monthly household income, education (≤elementary school, middle school, high school, or ≥college), smoking (non-smoker, past, or current smoker), alcohol drinking frequency (<1 or ≥1/month), walking (<30 or ≥30 min/day, 5 days/week), BMI (<25 or ≥25 kg/m^2^), menopause status, and comorbidities (doctor-diagnosed HTN, DM, or DL).

Based on the 2001 National Cholesterol Education Program/Adult Treatment Panel III [26] and the 2005 American Heart Association/National Heart, Lung, and Blood Institute [27] criteria, we defined MetS to occur when three or more of the following five factors were satisfied: (1) waist circumference ≥85 cm for women or ≥90 cm for men (Korean cutoff for abdominal obesity [28]); (2) serum triglyceride level ≥150 mg/dL or under treatment with drugs for DL; (3) serum high-density lipoprotein cholesterol level <50 mg/dL for women or <40 mg/dL for men; (4) blood pressure (BP) ≥130/85 mm Hg or under treatment with drugs for high BP; and (5) fasting plasma glucose level ≥100 mg/dL or under treatment with drugs for high glucose levels.

### 2.5. Statistical Analysis

We used STATA version 14.0 (StataCorp., College Station, TX, USA) for statistical analysis, and *p* < 0.05 was set as the statistical significance level. The KNHANES was conducted using a two-stage stratified cluster sampling method. Therefore, we assigned weights to the stratified data in our analysis.

Linear regression analyses, Mann–Whitney U tests, Kruskal–Wallis H tests, and χ^2^ tests were used to analyze and compare the participants’ general characteristics according to sex and group. Logistic regression analyses were performed to assess CVD risk factors according to group. Adjusted odds ratios (ORs) were derived after controlling for potential confounding variables, such as age, daily nutritional intake (total and fat), average monthly household income, education, occupation, smoking, alcohol drinking, walking, BMI status, comorbidities (doctor-diagnosed HTN, DM, or DL), sleep duration, and menopausal status (only in women). As a subgroup analysis, logistic regression analyses were also performed, stratifying by age (<50 or ≥50 years) and occupation (yes or no).

## 3. Results

### 3.1. General Characteristics by Sex

Table 1 shows the participants’ general characteristics according to their sex. The average age of the 16,121 participants was 48.11 years, and 58.17% were women. Participants were categorized into early sleep (those who went to bed before 23:17) and late sleep (those who went to bed at or after 23:17) groups based on the median bedtime.

The median MSF was 3.25 h (IQR 2.00 to 4.25) in men and 3.50 h (IQR 2.50 to 4.50) in women. The median MSFsc was 3.00 h (IQR 2.00 to 4.00) in men and 3.17 h (IQR 2.25 to 4.00) in women. The mean sleep duration was 7.18 ± 0.021 h in men and 7.22 ± 0.019 h in women. The proportion of infrequent breakfast eaters (0 times a week) was higher in men compared to women. Total energy and protein intake and average monthly household income were higher, and carbohydrate intake was lower, in men compared to women. Furthermore, the proportion of participants with an occupation, highly educated participants (≥college), current smokers, alcohol drinkers (≥1/month), participants with BMI ≥25 kg/m^2^, and participants with HTN, DM, and MetS were higher, and the proportion of participants with DL was lower, in men compared to women.

### 3.2. General Characteristics by Group

Table 2 shows the participants’ general characteristics according to group in men. Age was highest in group 1 (60.14 ± 0.33 years), and lowest in group 4 (35.87 ± 0.37 years). MSF and MSFsc were latest in group 4, and sleep duration was longest in group 3.

Carbohydrate intake and the proportion of participants with HTN, DM, DL and without an occupation were the highest in group 1. Protein intake, average monthly household income, the proportion of highly educated participants (≥college) and participants with BMI ≥25 kg/m^2^ were lowest in group 1.

Average monthly household income and the proportion of participants walking over 30 min ∗ 5 days/week were highest in group 2. 

The proportion of participants with an occupation, current smokers, alcohol drinkers (≥1/month), participants walking under 30 min ∗ 5 days, and participants with MetS were highest in group 3.

Protein and fat intake were highest in group 4. Also, the proportion of highly educated participants (≥college), non-smokers, and participants with BMI ≥25 kg/m^2^ were highest in group 4.

Table 3 shows the participants’ general characteristics according to group in women. As in men, age was highest in group 1 (60.56 ± 0.33 years), and lowest in group 4 (36.70 ± 0.35 years). MSF and MSFsc were also latest in group 4. Equally, sleep duration was longest in group 3 and shortest in group 2.

Likewise, carbohydrate intake and the proportion of participants with HTN, DM, DL and without an occupation were highest in group 1. On the other hand, in women, the proportion of non-smokers, participants with BMI ≥25 kg/m^2^, and participants with MetS were highest in group 1. Protein intake, average monthly household income, and the proportion of highly educated participants (≥college) were lowest in group 1.

Just as in men, average monthly household income and the proportion of participants walking over 30 min ∗ 5 days/week were highest in group 2. The proportion of participants with an occupation and walking under 30 min ∗ 5 days were highest in group 3.

Protein intake, fat intake, and the proportion of highly educated participants (≥college) were also highest in group 4. On the other hand, the proportion of current smokers and alcohol drinkers (≥1/month) were highest in group 4 for women, whereas these were highest in group 3 for men.

### 3.3. Cardiovascular Disease Risk Factors according to Breakfast Frequency and Sleep Type

Table 4 shows ORs for CVD risk factors according to breakfast frequency (5–7/week vs. 0–2/week) and sleep type (early sleep vs. late sleep).

After controlling for potential confounding variables, in men, group 4 (late sleep + infrequent breakfast eaters) had a lower prevalence of obesity than group 1 (early sleep + regular breakfast eaters) (OR 0.78, 95% confidence interval (CI) 0.62–0.97), and groups 2, 3, and 4 had a higher prevalence of MetS than group 1 (OR 1.43, 1.62, and 1.47, respectively).

In women, group 4 had a lower prevalence of DL than group 1 (OR 0.59, 95% CI 0.44–0.80), and group 2 had a higher prevalence of MetS than group 1 (OR 1.24, 95% CI 1.03–1.50).

### 3.4. Subgroup Analysis

Table 5 shows ORs for CVD risk factors according to breakfast frequency (5–7/week vs. 0–2/week) and sleep type (early sleep vs. late sleep) by age.

After controlling for potential confounding variables and stratifying based on age 50, in men under 50, group 3 (OR 1.83, 95% CI 1.15–2.93) and group 4 (OR 1.54, 95% CI 1.04–2.28) had a higher prevalence of MetS than group 1. In men over 50, group 2 (OR 1.51, 95% CI 1.20–1.90) and group 4 (OR 1.52, 95% CI 1.01–2.27) had a higher prevalence of MetS than group 1.

In women under 50, group 2 had a lower prevalence of obesity than group 1 (OR 0.71, 95% CI 0.52–0.96). In women over 50, group 4 had a higher prevalence of obesity (OR 1.55, 95% CI 1.12–2.14) and MetS (OR 1.68, 95% CI 1.19–2.39) and a lower prevalence of DL than group 1 (OR 0.67, 95% CI 0.47–0.94).

Table 6 shows ORs for CVD risk factors according to breakfast frequency (5–7/week vs. 0–2/week) and sleep type (early sleep vs. late sleep) by occupation.

After controlling for potential confounding variables, only in men who are employed, groups 2 and 4 had a lower prevalence of obesity than group 1 (OR 0.73, 95% CI 0.59–0.90 and OR 0.76, 95% CI 0.59–0.98, respectively). Moreover, group 3 had a lower prevalence of DM than group 1 (OR 0.44, 95% CI 0.21–0.96), and had a higher prevalence of MetS than group 1 (OR 1.69, 95% CI 1.21–2.36). In both unemployed and employed men, groups 2 and 4 had a higher prevalence of MetS than group 1.

In unemployed women, groups 2 (OR 1.39, 95% CI 1.09–1.79) and 4 (OR 1.59, 95% CI 1.09–2.31) had a higher prevalence of MetS than group 1. In employed women, groups 3 (OR 0.57, 95% CI 0.33–0.98) and 4 (OR 0.43, 95% CI 0.27–0.67) had a lower prevalence of MetS than group 1.

## 4. Discussion

To the best of our knowledge, this study is the first to examine the joint association of breakfast eating behavior and sleep timing with CVD risk factors. The results of our study indicate that the combined relationship between skipping breakfast and late sleep was associated with a higher prevalence of MetS after adjusting confounding variables including sleep duration, particularly in men. However, no significant or inverse association was found between the joint effect of breakfast eating behavior and sleep timing with CVD risk factors including obesity, DM, HTN, and DL.

Although previous studies have examined the individual impact of breakfast and sleep on MetS, their combined relationship remains understudied, with a primary focus on sleep duration [29,30]. For example, a cross-sectional study of Korean adults found that individuals who skipped breakfast and had short sleep duration (<6 h) had a higher risk of MetS [29]. However, this study only assessed breakfast intake during the previous two days and sleep duration, not sleep timing. Another recent cross-sectional study of Japanese adults found that both individual and joint association of skipping breakfast and short sleep duration (<6 h) were associated with a higher prevalence of MetS in men [30]. Likewise, our study revealed that the joint association of skipping breakfast and late sleep was associated with a higher prevalence of MetS in men compared to the reference group (regular breakfast and early sleep). In a subgroup analysis, this significant association persisted in men regardless of age group. In women, the group aged 50 years or over showed a significantly higher prevalence of MetS. Since the combined effect of breakfast and sleep timing has rarely been examined, there is uncertainty regarding the specific physiological mechanisms underlying these relationships. However, it has been suggested that both skipping breakfast and late sleep lead to a nocturnal lifestyle, characterized by late dinner, daytime sleepiness, and lower physical activity, which can disrupt regulating appetite, satiety, and glucose metabolism [31,32].

In contrast to the joint association of skipping breakfast and late sleep with MetS, our study found inverse relationships between these factors and obesity in men. Our findings differ from a previous study that found the combination of skipping breakfast and insufficient sleep was associated with a higher risk of obesity in children and adolescents [33]. However, to our knowledge, no study explored the association of breakfast and sleep timing with obesity in adults. Previous research has centered on investigating the separate association of breakfast and sleep timing with obesity, and the results have been inconclusive. Several prospective studies and a meta-analysis of 19 cross-sectional studies in Asian and Pacific regions revealed skipping breakfast was linked with an increased risk of obesity [4,34]. However, two recent meta-analyses of randomized controlled trials have shown that skipping breakfast was more closely associated with moderate weight loss than breakfast intake [35,36].

Similarly, there has been controversy in the literature regarding the association between sleep timing and obesity depending on sex and age group. For example, a multinational cross-sectional study found that later bedtimes (>10 PM) were associated with a higher prevalence of obesity compared with a bedtime between 8 to 10 PM [13]. Another study by Sasaki et al. found that the association between a late bedtime (24:00 h or later) and obesity remained significant among those aged over 65 years but not among younger individuals [11]. However, when the data were stratified by gender, neither men nor women showed a significant association. Additionally, Knutson et al. reported that a later bedtime was linked to lower BMI in those under 35 years, but the opposite association was observed among those aged 36–70 years [14]. Likewise, our findings varied depending on gender and age. Among men, the group who skipped breakfast and had late sleep had a lower prevalence of obesity. However, this association did not reach statistical significance when the data were analyzed based on age groups. Among women, no significant differences in the prevalence of obesity were found between the different sleep and breakfast groups in overall analysis. However, in women aged 50 years or older, the combination of skipping breakfast and late sleep was significantly associated with a higher prevalence of obesity. The reason for these mixed results is not clear and may be attributed to complex interactions between breakfast intake and sleep timing. These findings suggest that the combined effect of breakfast intake and sleep timing on obesity may vary across different sex and age groups.

Despite the widely established roles of breakfast and sleep in metabolic health, our study did not find any significant associations between their combined effects and the prevalence of DM and HTN across all age and sex groups. Previous research has solely examined the links between breakfast and sleep timing with DM and HTN separately. While a meta-analysis of prospective and cross-sectional studies found a positive association between skipping breakfast and DM, most of the individual studies have shown no significant association [37,38,39,40]. For example, Nakajima et al. examined the individual relationships of late-night dinner-eating (LNDE) and breakfast with hyperglycemia and revealed that breakfast alone was not associated with a higher blood glucose level, only LNDE [40]. Additionally, in the review article, the author presented opposing perspectives on breakfast consumption [41]. One viewpoint suggests that having breakfast in the morning is crucial for maintaining a healthy level of physical activity, while the other perspective indicates that consuming breakfast without a sufficient fasting may lead to sustained high blood glucose and increase insulin secretion.

With respect to sleep timing and DM and HTN, a prospective study of a 12-year follow-up of middle-aged Korean participants found that late sleep has been associated with a higher risk of DM [15]. However, the study participants were divided into three groups including early sleepers (22:00–22:59), usual sleepers (23:00–00:59), and late sleepers (1:00–5:59), and there was no significant difference between early and usual sleepers. Another study conducted among Mexican adolescents showed that those with bedtimes after 10 PM had a higher incidence of high blood pressure compared to those with bedtimes between 9 to 10 PM on weekdays over a 14-month follow-up period [17]. The observed discrepancies from our study were likely due to several factors, such as lack of consideration for breakfast, differences in participant characteristics and age, and the use of different categories for sleep timing. Specifically, our study used a median split as a cut-off point for analysis, while other studies categorized several specific time frames.

The relationship between breakfast and sleep timing and DL compared to other CVD risk factors has not been extensively studied. In our study, except for women who skipped breakfast and had late sleep, no significant differences were observed between the reference group (regular breakfast and early sleep) and the other groups. Although previous studies have shown that skipping breakfast is associated with a higher risk of dyslipidemia [42], the relationship between sleep and dyslipidemia has not fully explored, with most studies focusing on the association with obstructive sleep apnea [43,44]. Future studies are needed to examine the relationship between sleep timing and dyslipidemia, and the combined effect of skipping breakfast and sleep timing on dyslipidemia.

Furthermore, our study examined whether the combined relationships between breakfast and sleep timing with CVD risk factors differed based on occupational status (employed vs. unemployed). In modern society, most workers experience social jetlag, a mismatch between internal body clocks and social time determined by social obligations such as work. Considering that this misalignment can alter breakfast and sleep patterns, it is important to investigate how occupational status influences these associations. Our study found that, in men, late sleep groups compared to the reference group (early sleep and regular breakfast) had a higher prevalence of MetS regardless of occupational status, while in women this relationship was found only in the unemployed status. Furthermore, compared to the reference group, certain combinations of breakfast and sleep timing were associated with a lower risk of CVD risk factors (obesity and DM in men and DL in women) only in the employed groups. These results suggest that breakfast and sleep timing interact differently according to employment status and gender. Since previous studies have mainly focused on comparing workers and non-workers, limited research has compared groups based on their sleep and breakfast behaviors within each group. Therefore, it is difficult to identify clear mechanisms behind these findings. Future research is needed to determine whether working status acts as a moderating factor in the relationship between sleep and breakfast, and whether differences exist depending on gender.

The mechanisms underlying between sex- and age-specific breakfast and sleep patterns and CVD risk factors remains unclear. Our findings, consistent with our hypothesis, showed that skipping breakfast and late sleep were associated with a higher risk of MetS in men and women aged 50 years and older compared to the reference group. However, other than that, the results indicating significant differences between groups were not consistent across sex and age groups. Previous studies have shown that women tend to have an earlier circadian chronotype and are more susceptible to circadian rhythm misalignment than men [45,46]. Older adults tend to become morning types due to physiological changes in their bodies (e.g., changes in circadian rhythm, changes in melatonin and cortisol levels, weakness in the suprachiasmatic nucleus) [47,48]. These findings imply that circadian chronotype and physiological responses to circadian misalignment may vary across age and gender. In addition, our study showed unexpected inverse relationships, particularly between obesity and MetS in men. The reasons for the controversial findings are not clear, but certain attributes of our subjects may help explain these differences. Despite differences in breakfast and sleep patterns between the four groups, total energy intake (kcal/day) was similar. This suggests that the combination of skipping breakfast and late sleep does not necessarily lead to an increased total caloric intake. Other confounding factors, such as the quality of diet and sleep, could also have influenced these findings. Additionally, the group which skipped breakfast and had late sleep was the youngest compared to the other groups. Given the established relationship that basal metabolic rate generally declines with age [49], the higher metabolic rate in these young participants may offset potential weight gain and explain the observed lower prevalence of obesity. Future research is warranted to further elucidate the complex relationships between sex- and age-specific patterns in breakfast and sleep timing and their impact on metabolic and cardiovascular outcomes.

Despite the strengths of our study in investigating the joint association of breakfast consumption and sleep timing with CVD risk factors in all sex-by-age groups, there are some limitations to note. First, this is a cross-sectional study using the KNHANES data. Therefore, it is not possible to determine causality or examine the effects of changing sleep timing or breakfast consumption. Further research is needed to investigate the individual and joint effects of actual breakfast consumption and sleep timing on health outcomes through studies in a real-world setting. Second, the data on breakfast and sleep timing were collected through self-reported data, which would lead to recall bias and subjectivity. In addition, differences in the perceived standard of breakfast between subjects and differences in the actual and reported duration of sleep can lead to misclassification. Third, the current study lacked detailed information on the quality of diet and sleep which may have limited the accuracy of the findings. Including measures of diet quality and sleep quality in future studies would provide a more comprehensive understanding of the relationship between breakfast and sleep timing on CVD risk factors.

## 5. Conclusions

In conclusion, this study contributes to the understanding of the joint association between breakfast eating behavior and sleep timing on CVD risk factors. The combination of skipping breakfast and late sleep timing was found to be associated with a higher prevalence of MetS, particularly in men. Moreover, the relationship between breakfast and sleep timing on CVD risk factors differed by sex and age group. Further research is warranted to explore the associations between breakfast and sleep timing on CVD risk factors across different sex and age groups.

## Figures and Tables

**Table 1:** General characteristics of the participants by sex.

	Total (*n* = 16,121)	Men (*n* = 6744)	Women (*n* = 9377)	*p* Value
Age, years	48.11 ± 0.23	47.37 ± 0.28	48.84 ± 0.26	<0.001
Breakfast frequency				0.014
5–7/week	10,926 (67.77)	4589 (68.05)	6337 (67.58)	
3–4/week	1551 (9.62)	594 (8.81)	957 (10.21)	
1–2/week	1612 (10.00)	676 (10.02)	936 (9.98)	
0/week	2032 (12.60)	885 (13.12)	1147 (12.23)	
Sleep type				<0.001
Late (bedtime ≥23:17)	8154 (50.58)	3284 (48.70)	4870 (51.94)	
Early (bedtime <23:17)	7967 (49.42)	3460 (51.30)	4507 (48.06)	
MSF, hours (median (IQR))	3.25 (2.25 to 4.38)	3.25 (2.00 to 4.25)	3.50 (2.50 to 4.50)	<0.001
MSFsc, hours (median (IQR))	3.11 (2.14 to 4.00)	3.00 (2.00 to 4.00)	3.17 (2.25 to 4.00)	<0.001
Group				<0.001
Early sleep + regular breakfast	6592 (45.24)	2894 (47.06)	3698 (43.92)	
Late sleep + regular breakfast	4334 (29.75)	1695 (27.56)	2639 (31.34)	
Early sleep + infrequent breakfast	878 (6.03)	389 (6.33)	489 (5.81)	
Late sleep + infrequent breakfast	2766 (18.98)	1172 (19.06)	1594 (18.93)	
Sleep duration, hours	7.20 ± 0.015	7.18 ± 0.021	7.22±0.019	0.168
Nutritional intake				
Total energy intake, kcal/day	1947.58±9.19	2251.83 ± 13.17	1650.97 ± 8.96	<0.001
Carbohydrates, % of energy	61.18 ± 0.15	59.09 ± 0.21	63.21 ± 0.17	<0.001
Protein, % of energy	14.63 ± 0.050	14.78 ± 0.071	14.49 ± 0.058	<0.001
Fat, % of energy	20.28 ± 0.11	20.14 ± 0.15	20.43 ± 0.13	0.077
Average monthly household income, 10,000 KRW	463.48 ± 6.72	472.69 ± 7.32	454.51 ± 7.19	0.001
Occupation				<0.001
No	6548 (40.62)	2008 (29.77)	4540 (48.42)	
Yes	9573 (59.38)	4736 (70.23)	4837 (51.58)	
Education				<0.001
≤Elementary school	3501 (21.74)	1063 (15.77)	2438 (26.04)	
Middle school	1695 (10.53)	729 (10.82)	966 (10.32)	
High school	5042 (31.31)	2296 (34.07)	2746 (29.33)	
≥College	5866 (36.43)	2652 (39.35)	3214 (34.32)	
Smoking				<0.001
None	10,409 (64.70)	1865 (27.70)	8544 (91.34)	
Past	3192 (19.84)	2779 (41.27)	413 (4.42)	
Current	2486 (15.45)	2089 (31.03)	397 (4.24)	
Alcohol drinking				<0.001
<1 time/month	7849 (48.77)	2150 (31.92)	5699 (60.90)	
≥1 time/month	8245 (51.23)	4586 (68.08)	3659 (39.10)	
Walking				0.416
<30 min ∗ 5 days/week	9881 (61.45)	4111 (61.08)	5770 (61.72)	
≥30 min ∗ 5 days/week	6198 (38.55)	2619 (38.92)	3579 (38.28)	
Body mass index				<0.001
<25 kg/m^2^	10,384 (64.81)	3952 (58.91)	6432 (69.06)	
≥25 kg/m^2^	5638 (35.19)	2756 (41.09)	2882 (30.94)	
Hypertension	4262 (26.44)	1933 (28.66)	2329 (24.84)	<0.001
Diabetes	1681 (10.43)	801 (11.88)	880 (9.38)	<0.001
Dyslipidemia	3333 (20.67)	1213 (17.99)	2120 (22.61)	<0.001
Metabolic syndrome	4243 (26.32)	2048 (30.37)	2195 (23.41)	<0.001

MSF: mid-sleep on free days; MSFsc: mid-sleep on free days corrected for sleep debt on work days; IQR: Interquartile range; KRW: Korea republic won.; Data are presented as mean ± standard error or median (interquartile range) for continuous variables (linear regression or Mann-Whitney U test) and as numbers (%) for categorical variables (χ^2^ test).

**Table 2:** General characteristics of the participants by group in men.

	Early Sleep + Regular Breakfast (*n* = 2894)	Late Sleep + Regular Breakfast (*n* = 1695)	Early Sleep + Infrequent Breakfast (*n* = 389)	Late Sleep + Infrequent Breakfast (*n* = 1172)	*p* Value
Age, years	60.14 ± 0.33	46.35 ± 0.43	44.99 ± 0.70	35.87 ± 0.37	<0.001
MSF, hours (median (IQR))	2.00 (1.50 to 2.75)	3.75 (3.25 to 4.50)	2.75 (2.00 to 3.50)	5.00 (4.00 to 6.00)	<0.001
MSFsc, hours (median (IQR))	2.00 (1.50 to 2.64)	3.57 (3.10 to 4.14)	2.54 (2.00 to 3.14)	4.46 (3.64 to 5.64)	<0.001
Sleep duration, hours	7.58 ± 0.028	6.72 ± 0.034	7.84 ± 0.080	7.04 ± 0.045	<0.001
Nutritional intake					
Total energy intake, kcal/day	1876.85 ± 12.82	2014.85 ± 16.05	1903.57 ± 30.75	1933.54 ± 18.92	0.227
Carbohydrates, % of energy	65.85 ± 0.19	61.14 ± 0.23	58.75 ± 0.58	56.41 ± 0.32	<0.001
Protein, % of energy	14.08 ± 0.068	14.88 ± 0.086	14.38 ± 0.17	15.08 ± 0.11	<0.001
Fat, % of energy	17.03 ± 0.14	20.71 ± 0.17	20.54 ± 0.37	23.31 ± 0.23	<0.001
Average monthly household income, 10,000 KRW	387.21 ± 7.51	513.48 ± 8.47	476.97 ± 13.16	494.63 ± 10.72	<0.001
Occupation					<0.001
No	1083 (37.42)	455 (26.84)	58 (14.91)	278 (23.72)	
Yes	1811 (62.58)	1240 (73.16)	331 (85.09)	894 (76.28)	
Education					<0.001
≤Elementary school	824 (28.50)	136 (8.02)	40 (10.28)	24 (2.05)	
High school	482 (16.67)	129 (7.61)	41 (10.54)	43 (3.67)	
Middle school	868 (30.02)	580 (34.22)	139 (35.73)	485 (41.38)	
≥College	717 (24.80)	850 (50.15)	169 (43.44)	620 (52.90)	
Smoking					<0.001
None	724 (25.10)	502 (29.65)	68 (17.48)	372 (31.74)	
Past	1523 (52.79)	680 (40.17)	115 (29.56)	289 (24.66)	
Current	638 (22.11)	511 (30.18)	206 (52.96)	511 (43.60)	
Alcohol drinking					<0.001
<1 time/month	1071 (37.10)	530 (31.29)	90 (23.14)	296 (25.26)	
≥1 time/month	1816 (62.90)	1164 (68.71)	299 (76.86)	876 (74.74)	
Walking					0.007
<30 min ∗ 5 days/week	1759 (60.99)	998 (58.91)	253 (65.21)	756 (64.62)	
≥30 min ∗ 5 days/week	1125 (39.01)	696 (41.09)	135 (34.79)	414 (35.38)	
Body mass index					<0.001
<25 kg/m^2^	1770 (61.61)	993 (58.86)	221 (56.96)	621 (53.21)	
≥25 kg/m^2^	1103 (38.39)	694 (41.14)	167 (43.04)	546 (46.79)	
Hypertension	1254 (43.33)	399 (23.54)	74 (19.02)	123 (10.49)	<0.001
Diabetes	526 (18.18)	182 (10.74)	25 (6.43)	40 (3.41)	<0.001
Dyslipidemia	704 (24.33)	298 (17.58)	58 (14.91)	97 (8.28)	<0.001
Metabolic syndrome	941 (32.52)	509 (30.03)	136 (34.96)	314 (26.79)	0.001

IQR: Interquartile range; KRW: Korea republic won. Data are presented as mean ± standard error or median (interquartile range) for continuous variables (linear regression or Kruskal–Wallis H test) and as numbers (%) for categorical variables (χ^2^ test).

**Table 3:** General characteristics of the participants by group in women.

	Early Sleep + Regular Breakfast (*n* = 3698)	Late Sleep + Regular Breakfast (*n* = 2639)	Early Sleep + Infrequent Breakfast (*n* = 489)	Late Sleep + Infrequent Breakfast (*n* = 1594)	*p* Value
Age, years	60.56 ± 0.33	48.70 ± 0.36	45.42 ± 0.78	36.70 ± 0.35	<0.001
MSF, hours (median (IQR))	2.25 (1.50 to 3.00)	4.00 (3.33 to 4.50)	3.00 (2.25 to 3.50)	4.75 (4.00 to 6.00)	<0.001
MSFsc, hours (median (IQR))	2.11 (1.50 to 2.79)	3.64 (3.14 to 4.14)	2.79 (2.14 to 3.35)	4.36 (3.64 to 5.43)	<0.001
Sleep duration, hours	7.62 ± 0.027	6.69 ± 0.028	8.15 ± 0.072	7.07 ± 0.041	<0.001
Nutritional intake					
Total energy intake, kcal/day	1876.85 ± 12.82	2014.85 ± 16.05	1903.57 ± 30.75	1933.54 ± 18.92	0.663
Carbohydrates, % of energy	65.85 ± 0.19	61.14 ± 0.23	58.75 ± 0.58	56.41 ± 0.32	<0.001
Protein, % of energy	14.08 ± 0.068	14.88 ± 0.086	14.38 ± 0.17	15.08 ± 0.11	<0.001
Fat, % of energy	17.03 ± 0.14	20.71 ± 0.17	20.54 ± 0.37	23.31 ± 0.23	<0.001
Average monthly household income, 10,000 KRW	387.21 ± 7.51	513.48 ± 8.47	476.97 ± 13.16	494.63 ± 10.72	<0.001
Occupation					<0.001
No	2007 (54.27)	1302 (49.34)	167 (34.15)	627 (39.34)	
Yes	1691 (45.73)	1337 (50.66)	322 (65.85)	967 (60.66)	
Education					<0.001
≤Elementary school	1734 (46.98)	460 (17.46)	74 (15.13)	71 (4.46)	
Middle school	527 (14.28)	238 (9.04)	45 (9.20)	88 (5.52)	
High school	800 (21.67)	860 (32.65)	156 (31.90)	616 (38.67)	
≥College	630 (17.07)	1076 (40.85)	214 (43.76)	818 (51.35)	
Smoking					<0.001
None	3522 (95.63)	2433 (92.37)	429 (87.73)	1310 (82.23)	
Past	91 (2.47)	110 (4.18)	31 (6.34)	120 (7.53)	
Current	70 (1.90)	91 (3.45)	29 (5.93)	163 (10.23)	
Alcohol drinking					<0.001
<1 time/month	2703 (73.33)	1583 (60.10)	257 (52.56)	696 (43.69)	
≥1 time/month	983 (26.67)	1051 (39.90)	232 (47.44)	897 (56.31)	
Walking					<0.001
<30 minute ∗ 5 days/week	2310 (62.81)	1520 (57.66)	321 (65.78)	1020 (64.03)	
≥30 minute ∗ 5 days/week	1368 (37.19)	1116 (42.34)	167 (34.22)	573 (35.97)	
Body mass index					<0.001
<25 kg/m^2^	2352 (64.26)	1885 (71.86)	347 (71.11)	1161 (72.97)	
≥25 kg/m^2^	1308 (35.74)	738 (28.14)	141 (28.89)	430 (27.03)	
Hypertension	1495 (40.43)	540 (20.46)	65 (13.29)	117 (7.34)	<0.001
Diabetes	539 (14.58)	243 (9.21)	28 (5.73)	33 (2.07)	<0.001
Dyslipidemia	1241 (33.56)	554 (20.99)	68 (13.91)	131 (8.22)	<0.001
Metabolic syndrome	1162 (31.43)	570 (21.60)	94 (19.22)	226 (14.18)	<0.001

IQR: Interquartile range; KRW: Korea republic won. Data are presented as mean ± standard error or median (interquartile range) for continuous variables (linear regression or Kruskal–Wallis H test) and as numbers (%) for categorical variables (χ^2^ test).

**Table 4:** Multivariable logistic regression for cardiovascular disease risk factors according to the breakfast frequency (5–7/week vs. 0–2/week) and the sleep type (early sleep vs. late sleep).

	Crude	Age Adjusted	Multivariable
Men			
Obesity			
Early sleep + regular breakfast	reference	reference	reference
Late sleep + regular breakfast	1.12 (0.99 to 1.27)	0.95 (0.83 to 1.09)	0.78 (0.65 to 0.94)
Early sleep + infrequent breakfast	1.21 (0.98 to 1.50)	0.99 (0.79 to 1.24)	0.88 (0.66 to 1.18)
Late sleep + infrequent breakfast	1.41 (1.23 to 1.62)	1.03 (0.87 to 1.22)	0.78 (0.62 to 0.97)
Hypertension			
Early sleep + regular breakfast	reference	reference	reference
Late sleep + regular breakfast	0.40 (0.35 to 0.46)	0.87 (0.75 to 1.02)	0.90 (0.73 to 1.11)
Early sleep + infrequent breakfast	0.31 (0.24 to 0.40)	0.84 (0.62 to 1.12)	0.90 (0.62 to 1.31)
Late sleep + infrequent breakfast	0.15 (0.13 to 0.19)	0.80 (0.63 to 1.01)	0.98 (0.72 to 1.32)
Diabetes			
Early sleep + regular breakfast	reference	reference	reference
Late sleep + regular breakfast	0.54 (0.45 to 0.65)	1.03 (0.85 to 1.26)	1.10 (0.84 to 1.44)
Early sleep + infrequent breakfast	0.31 (0.20 to 0.47)	0.71 (0.46 to 1.09)	0.58 (0.32 to 1.05)
Late sleep + infrequent breakfast	0.16 (0.11 to 0.22)	0.63 (0.44 to 0.91)	0.77 (0.49 to 1.21)
Dyslipidemia			
Early sleep + regular breakfast	reference	reference	reference
Late sleep + regular breakfast	0.66 (0.57 to 0.77)	1.04 (0.88 to 1.23)	0.93 (0.75 to 1.16)
Early sleep + infrequent breakfast	0.55 (0.41 to 0.73)	0.96 (0.71 to 1.30)	0.94 (0.63 to 1.41)
Late sleep + infrequent breakfast	0.28 (0.22 to 0.35)	0.70 (0.54 to 0.91)	0.75 (0.54 to 1.04)
Metabolic syndrome			
Early sleep + regular breakfast	reference	reference	reference
Late sleep + regular breakfast	0.89 (0.78 to 1.01)	1.15 (0.996 to 1.32)	1.43 (1.18 to 1.74)
Early sleep + infrequent breakfast	1.12 (0.89 to 1.39)	1.52 (1.21 to 1.93)	1.62 (1.20 to 2.20)
Late sleep + infrequent breakfast	0.76 (0.65 to 0.88)	1.24 (1.03 to 1.50)	1.47 (1.15 to 1.88)
Women			
Obesity			
Early sleep + regular breakfast	reference	reference	reference
Late sleep + regular breakfast	0.70 (0.63 to 0.78)	0.94 (0.84 to 1.05)	0.93 (0.79 to 1.09)
Early sleep + infrequent breakfast	0.73 (0.59 to 0.90)	1.09 (0.88 to 1.35)	1.09 (0.82 to 1.45)
Late sleep + infrequent breakfast	0.67 (0.58 to 0.76)	1.23 (1.05 to 1.43)	1.10 (0.89 to 1.35)
Hypertension			
Early sleep + regular breakfast	reference	reference	reference
Late sleep + regular breakfast	0.38 (0.34 to 0.43)	0.89 (0.78 to 1.02)	0.89 (0.73 to 1.07)
Early sleep + infrequent breakfast	0.23 (0.17 to 0.30)	0.70 (0.51 to 0.96)	0.81 (0.54 to 1.22)
Late sleep + infrequent breakfast	0.12 (0.10 to 0.14)	0.80 (0.63 to 1.01)	0.83 (0.61 to 1.14)
Diabetes			
Early sleep + regular breakfast	reference	reference	reference
Late sleep + regular breakfast	0.59 (0.51 to 0.70)	1.20 (1.01 to 1.43)	1.15 (0.90 to 1.46)
Early sleep + infrequent breakfast	0.36 (0.24 to 0.53)	0.92 (0.61 to 1.40)	0.99 (0.58 to 1.70)
Late sleep + infrequent breakfast	0.12 (0.087 to 0.18)	0.60 (0.41 to 0.88)	0.72 (0.45 to 1.16)
Dyslipidemia			
Early sleep + regular breakfast	reference	reference	reference
Late sleep + regular breakfast	0.53 (0.47 to 0.59)	1.04 (0.88 to 1.23)	0.91 (0.76 to 1.09)
Early sleep + infrequent breakfast	0.32 (0.25 to 0.42)	0.96 (0.71 to 1.30)	0.75 (0.51 to 1.10)
Late sleep + infrequent breakfast	0.18 (0.15 to 0.21)	0.70 (0.54 to 0.91)	0.59 (0.44 to 0.80)
Metabolic syndrome			
Early sleep + regular breakfast	reference	reference	reference
Late sleep + regular breakfast	0.60 (0.54 to 0.67)	1.03 (0.91 to 1.17)	1.24 (1.03 to 1.50)
Early sleep + infrequent breakfast	0.52 (0.41 to 0.66)	1.11 (0.86 to 1.43)	1.27 (0.90 to 1.79)
Late sleep + infrequent breakfast	0.36 (0.31 to 0.42)	1.17 (0.97 to 1.41)	1.22 (0.94 to 1.59)

**Table 5:** Multivariable logistic regression for cardiovascular disease risk factors according to the breakfast frequency (5–7/week vs. 0–2/week) and the sleep type (early sleep vs. late sleep) by age.

	<50 Years	≥50 Years
Men		
Obesity		
Early sleep + regular breakfast	reference	reference
Late sleep + regular breakfast	0.79 (0.57 to 1.10)	0.78 (0.63 to 0.98)
Early sleep + infrequent breakfast	0.88 (0.58 to 1.35)	0.77 (0.48 to 1.24)
Late sleep + infrequent breakfast	0.72 (0.52 to 1.005)	0.89 (0.60 to 1.33)
Hypertension		
Early sleep + regular breakfast	reference	reference
Late sleep + regular breakfast	1.17 (0.66 to 2.09)	0.86 (0.69 to 1.07)
Early sleep + infrequent breakfast	0.80 (0.37 to 1.74)	1.01 (0.64 to 1.58)
Late sleep + infrequent breakfast	1.12 (0.61 to 2.06)	1.10 (0.74 to 1.64)
Diabetes		
Early sleep + regular breakfast	reference	reference
Late sleep + regular breakfast	2.38 (0.72 to 7.81)	1.03 (0.78 to 1.37)
Early sleep + infrequent breakfast	0.42 (0.043 to 4.12)	0.70 (0.37 to 1.31)
Late sleep + infrequent breakfast	2.99 (0.85 to 10.50)	0.63 (0.35 to 1.13)
Dyslipidemia		
Early sleep + regular breakfast	reference	reference
Late sleep + regular breakfast	1.03 (0.58 to 1.84)	0.90 (0.71 to 1.15)
Early sleep + infrequent breakfast	1.24 (0.60 to 2.57)	0.78 (0.45 to 1.33)
Late sleep + infrequent breakfast	0.79 (0.42 to 1.49)	1.04 (0.68 to 1.60)
Metabolic syndrome		
Early sleep + regular breakfast	reference	reference
Late sleep + regular breakfast	1.13 (0.77 to 1.66)	1.51 (1.20 to 1.90)
Early sleep + infrequent breakfast	1.83 (1.15 to 2.93)	1.06 (0.66 to 1.69)
Late sleep + infrequent breakfast	1.54 (1.04 to 2.28)	1.52 (1.01 to 2.27)
Women		
Obesity		
Early sleep + regular breakfast	reference	reference
Late sleep + regular breakfast	0.71 (0.52 to 0.96)	0.99 (0.82 to 1.20)
Early sleep + infrequent breakfast	0.84 (0.55 to 1.28)	1.25 (0.81 to 1.93)
Late sleep + infrequent breakfast	0.86 (0.63 to 1.19)	1.55 (1.12 to 2.14)
Hypertension		
Early sleep + regular breakfast	reference	reference
Late sleep + regular breakfast	0.64 (0.32 to 1.30)	0.91 (0.74 to 1.11)
Early sleep + infrequent breakfast	0.71 (0.23 to 2.15)	0.86 (0.54 to 1.34)
Late sleep + infrequent breakfast	1.15 (0.54 to 2.45)	0.76 (0.53 to 1.09)
Diabetes		
Early sleep + regular breakfast	reference	reference
Late sleep + regular breakfast	0.51 (0.15 to 1.73)	1.18 (0.92 to 1.52)
Early sleep + infrequent breakfast	1.81 (0.39 to 8.36)	0.96 (0.53 to 1.72)
Late sleep + infrequent breakfast	0.51 (0.13 to 2.05)	0.71 (0.41 to 1.22)
Dyslipidemia		
Early sleep + regular breakfast	reference	reference
Late sleep + regular breakfast	1.04 (0.57 to 1.89)	0.89 (0.74 to 1.07)
Early sleep + infrequent breakfast	0.80 (0.34 to 1.89)	0.74 (0.47 to 1.14)
Late sleep + infrequent breakfast	0.65 (0.32 to 1.30)	0.67 (0.47 to 0.94)
Metabolic syndrome		
Early sleep + regular breakfast	reference	reference
Late sleep + regular breakfast	1.29 (0.84 to 1.99)	1.18 (0.96 to 1.45)
Early sleep + infrequent breakfast	1.18 (0.65 to 2.16)	1.43 (0.91 to 2.24)
Late sleep + infrequent breakfast	1.03 (0.64 to 1.65)	1.68 (1.19 to 2.39)

Data are presented as odds ratios with 95% confidence intervals. Multivariable model: adjusted for age, daily nutritional intake (total and fat), average monthly household income, education, occupation, smoking, alcohol drinking, walking, body mass index status, comorbidities (doctor-diagnosed hypertension, diabetes, or dyslipidemia), sleep duration, and menopausal status (only in women).

**Table 6:** Multivariable logistic regression for cardiovascular disease risk factors according to the breakfast frequency (5–7/week vs. 0–2/week) and the sleep type (early sleep vs. late sleep) by occupation.

	Unemployed	Employed
Men		
Obesity		
Early sleep + regular breakfast	reference	reference
Late sleep + regular breakfast	0.95 (0.67 to 1.35)	0.73 (0.59 to 0.90)
Early sleep + infrequent breakfast	0.63 (0.27 to 1.45)	0.90 (0.66 to 1.24)
Late sleep + infrequent breakfast	0.77 (0.47 to 1.27)	0.76 (0.59 to 0.98)
Hypertension		
Early sleep + regular breakfast	reference	reference
Late sleep + regular breakfast	0.93 (0.65 to 1.33)	0.90 (0.70 to 1.15)
Early sleep + infrequent breakfast	1.57 (0.69 to 3.55)	0.81 (0.53 to 1.24)
Late sleep + infrequent breakfast	0.98 (0.51 to 1.92)	0.99 (0.70 to 1.40)
Diabetes		
Early sleep + regular breakfast	reference	reference
Late sleep + regular breakfast	1.30 (0.84 to 2.00)	0.93 (0.65 to 1.31)
Early sleep + infrequent breakfast	0.95 (0.36 to 2.55)	0.44 (0.21 to 0.96)
Late sleep + infrequent breakfast	0.49 (0.17 to 1.39)	0.81 (0.48 to 1.35)
Dyslipidemia		
Early sleep + regular breakfast	reference	reference
Late sleep + regular breakfast	0.76 (0.51 to 1.13)	1.03 (0.79 to 1.35)
Early sleep + infrequent breakfast	0.79 (0.29 to 2.13)	1.02 (0.65 to 1.61)
Late sleep + infrequent breakfast	0.70 (0.34 to 1.43)	0.80 (0.55 to 1.16)
Metabolic syndrome		
Early sleep + regular breakfast	reference	reference
Late sleep + regular breakfast	1.49 (1.03 to 2.15)	1.41 (1.12 to 1.77)
Early sleep + infrequent breakfast	1.15 (0.51 to 2.56)	1.69 (1.21 to 2.36)
Late sleep + infrequent breakfast	1.85 (1.05 to 3.25)	1.41 (1.07 to 1.87)
Women		
Obesity		
Early sleep + regular breakfast	reference	reference
Late sleep + regular breakfast	0.84 (0.67 to 1.05)	1.02 (0.81 to 1.30)
Early sleep + infrequent breakfast	0.93 (0.58 to 1.50)	1.23 (0.85 to 1.76)
Late sleep + infrequent breakfast	0.90 (0.66 to 1.23)	1.30 (0.98 to 1.73)
Hypertension		
Early sleep + regular breakfast	reference	reference
Late sleep + regular breakfast	0.93 (0.72 to 1.19)	0.82 (0.61 to 1.11)
Early sleep + infrequent breakfast	0.63 (0.33 to 1.21)	1.02 (0.60 to 1.72)
Late sleep + infrequent breakfast	0.67 (0.42 to 1.06)	1.01 (0.65 to 1.55)
Diabetes		
Early sleep + regular breakfast	reference	reference
Late sleep + regular breakfast	1.19 (0.88 to 1.62)	1.05 (0.70 to 1.58)
Early sleep + infrequent breakfast	1.13 (0.54 to 2.37)	0.91 (0.40 to 2.06)
Late sleep + infrequent breakfast	0.88 (0.48 to 1.62)	0.56 (0.25 to 1.22)
Dyslipidemia		
Early sleep + regular breakfast	reference	reference
Late sleep + regular breakfast	0.92 (0.73 to 1.16)	0.93 (0.71 to 1.23)
Early sleep + infrequent breakfast	1.04 (0.59 to 1.83)	0.57 (0.33 to 0.98)
Late sleep + infrequent breakfast	0.86 (0.57 to 1.30)	0.43 (0.27 to 0.67)
Metabolic syndrome		
Early sleep + regular breakfast	reference	reference
Late sleep + regular breakfast	1.39 (1.09 to 1.79)	1.08 (0.82 to 1.44)
Early sleep + infrequent breakfast	1.59 (0.92 to 2.74)	1.07 (0.68 to 1.68)
Late sleep + infrequent breakfast	1.59 (1.09 to 2.31)	0.96 (0.66 to 1.38)

Data are presented as odds ratios with 95% confidence intervals. Multivariable model: adjusted for age, daily nutritional intake (total and fat), average monthly household income, education, occupation, smoking, alcohol drinking, walking, body mass index status, comorbidities (doctor-diagnosed hypertension, diabetes, or dyslipidemia), sleep duration, and menopausal status (only in women).

## Data Availability

The data presented in this study are publicly available in the Korea National Health and Nutrition Examination Survey database at https://knhanes.kdca.go.kr/knhanes/sub03/sub03_02_05.do (accessed on 13 September 2023) and https://knhanes.kdca.go.kr/knhanes/eng/index.do (accessed on 13 September 2023).
